# High Frequency Migraine Is Associated with Lower Acute Pain Sensitivity and Abnormal Insula Activity Related to Migraine Pain Intensity, Attack Frequency, and Pain Catastrophizing

**DOI:** 10.3389/fnhum.2016.00489

**Published:** 2016-09-29

**Authors:** Vani A. Mathur, Massieh Moayedi, Michael L. Keaser, Shariq A. Khan, Catherine S. Hubbard, Madhav Goyal, David A. Seminowicz

**Affiliations:** ^1^Department of Neural and Pain Sciences, University of Maryland School of DentistryBaltimore, MD, USA; ^2^Department of Psychology, Texas A&M UniversityCollege Station, TX, USA; ^3^Department of Psychiatry and Behavioral Sciences, Johns Hopkins University School of MedicineBaltimore, MD, USA; ^4^Faculty of Dentistry, University of TorontoToronto, ON, Canada; ^5^Department of Medicine at Johns Hopkins, Division of General Internal Medicine, Johns Hopkins School of MedicineBaltimore, MD, USA; ^6^Center to Advance Chronic Pain Research, University of Maryland BaltimoreBaltimore, MD, USA

**Keywords:** headache, fMRI, DTI, pain modulation, chronic pain, pain intensity

## Abstract

Migraine is a pain disorder associated with abnormal brain structure and function, yet the effect of migraine on acute pain processing remains unclear. It also remains unclear whether altered pain-related brain responses and related structural changes are associated with clinical migraine characteristics. Using fMRI and three levels of thermal stimuli (non-painful, mildly painful, and moderately painful), we compared whole-brain activity between 14 migraine patients and 14 matched controls. Although, there were no significant differences in pain thresholds nor in pre-scan pain ratings to mildly painful thermal stimuli, patients did have aberrant suprathreshold nociceptive processing. Brain imaging showed that, compared to controls, patients had reduced activity in pain modulatory regions including left dorsolateral prefrontal, posterior parietal, and middle temporal cortices and, at a lower-threshold, greater activation in the right mid-insula to moderate pain vs. mild pain. We also found that pain-related activity in the insula was associated with clinical variables in patients, including associations between: bilateral anterior insula and pain catastrophizing (PCS); bilateral anterior insula and contralateral posterior insula and migraine pain intensity; and bilateral posterior insula and migraine frequency at a lower-threshold. PCS and migraine pain intensity were also negatively associated with activity in midline regions including posterior cingulate and medial prefrontal cortices. Diffusion tensor imaging revealed a negative correlation between fractional anisotropy (a measure of white matter integrity; FA) and migraine duration in the right mid-insula and a positive correlation between left mid-insula FA and PCS. In sum, while patients showed lower sensitivity to acute noxious stimuli, the neuroimaging findings suggest enhanced nociceptive processing and significantly disrupted modulatory networks, particularly involving the insula, associated with indices of disease severity in migraine.

## Introduction

Migraine is a central nervous system disease associated with painful, debilitating headache attacks, and reduced quality of life (Stewart et al., 1992; Goadsby, 1997; Goadsby et al., 2002; Lipton and Pan, 2004; Dodick, 2008; Charles, 2009; Sprenger and Borsook, 2012). Recent studies suggest that migraine shares physiological mechanisms as well as long lasting clinical and sociodemographic profiles with other chronic pain disorders (Buse et al., 2010; Blumenfeld et al., 2011; Yoon et al., 2013; Boyer et al., 2014). Although specific brain mechanisms have yet to be identified, migraine appears to alter the structure (Davis and Moayedi, 2013), function (Maniyar and Goadsby, 2013), and neurochemistry (Prescot et al., 2009) of the trigeminal nociceptive system, as well as disrupting resting state networks (Russo et al., 2012a; Sprenger and Borsook, 2012; Xue et al., 2012; Lakhan et al., 2013; Noseda and Burstein, 2013; Tessitore et al., 2013, 2015; Hubbard et al., 2014; Zhao et al., 2014; Liu et al., 2015; Tedeschi et al., 2016).

Low-frequency migraine alters trigeminal somatosensory-related brain responses. For example, patients with migraine showed less function in modulatory brainstem and cortical regions to a nociceptive stimulus (Moulton et al., 2008; Aderjan et al., 2010; Stankewitz et al., 2011) and more activity in other cortical regions such as the temporal pole, compared to pain-free controls (Moulton et al., 2011). One study identified that innocuous air puffs elicited greater levels of activity in the trigeminal spinal nucleus, the hypothalamus, the putamen, the insula (INS), the secondary somatosensory cortex (S2), and the supramarginal gyrus. However, another study found nociceptive stimulation in the trigeminal region led to decreased activity in the bilateral S2 (Russo et al., 2012b). Additionally, one study found increased trigeminal pain-related activity in the pregenual anterior cingulate cortex (pACC) (Russo et al., 2012b), whereas another study found increased activity in the pACC after repeated nociceptive stimulation over 8 days in patients with migraine (Aderjan et al., 2010).

With increasing frequency of attacks, migraine becomes a more debilitating disease (Olesen, 2008). Therefore, migraine patients with a high frequency of attacks should exhibit greater central abnormalities. Indeed, one study found abnormal increases in laser-evoked potential amplitudes to increasing nociceptive stimulus intensity, suggesting abnormal central nociceptive processing in patients with higher attack frequency (de Tommaso et al., 2003). Nonetheless, most studies investigating functional abnormalities have focused on low frequency migraine. Here, we aim use fMRI to identify whether patients with high attack frequency have abnormal nociceptive processing to extra-trigeminal stimuli, compared to healthy controls.

A prevalent hypothesis is that migraine progressively drives maladaptive plasticity in the brain, and leads to the chronification of pain (Borsook et al., 2012). This concept of maladaptive plasticity as a facilitator of chronic pain is supported by the correlation between brain structural and functional abnormalities and indices of disease severity in migraine and other chronic pain populations. For example, previously reported correlations between migraine severity and pain-related brain responses (Stankewitz et al., 2011; Schwedt et al., 2014) support this hypothesis.

Another important factor affecting nociceptive processing in migraine is pain catastrophizing, a maladaptive coping strategy associated with increased rumination, magnification, and helplessness toward pain (Sullivan et al., 1995). Migraine patients catastrophize more than healthy controls (Hassinger et al., 1999), and pain catastrophizing is a significant predictor of migraine chronicity (Radat et al., 2009), severity, disability, and quality of life (Holroyd et al., 2007). Studies have demonstrated that pain catastrophizing modulates neural pain processing in healthy (Seminowicz and Davis, 2006) and fibromyalgia (Gracely et al., 2004) populations, but similar examinations have not been conducted in migraine patients.

In addition to changes in nociceptive-related processing, indices of disease severity and pain catastrophizing have been related to structural abnormalities in chronic pain disorders (Davis and Moayedi, 2013). Specifically, in migraine, gray matter abnormalities have been related to disease duration (Rocca et al., 2006; Kim et al., 2008; Hubbard et al., 2014) and headache pain intensity and/or frequency (Kim et al., 2008; Valfrè et al., 2008; Hubbard et al., 2014). However, such relationships with indices of white matter integrity have yet to be investigated.

The relationship between altered pain-related brain responses in high-frequency migraine and clinical characteristics, such as migraine severity and pain catastrophizing remain unclear. While migraine headaches clearly involve sensitization of the trigeminal nociceptive system, allodynia, and muscle tenderness experienced outside the trigeminal area is common (Burstein et al., 2015), suggesting the presence of generalized central sensitization and aberrant nociceptive processing in migraine patients. Here, we examine the structure and function of pain-related brain regions in migraine patients and healthy controls, and evaluate the effects of indices of disease severity (migraine pain intensity, frequency, and duration) and pain catastrophizing. We hypothesized that migraine patients would have abnormal brain responses to nociceptive stimuli outside the trigeminal system, indicative of central sensitization (increased activity in pain-related areas Duerden and Albanese, 2013) and decreased activity in pain modulatory circuits including dorsolateral prefrontal cortex (DLPFC) and the periaqueductal gray (for a comprehensive review of modulatory brain networks, see: Tracey and Mantyh, 2007; Moayedi and Salomons, 2016). We further hypothesized these functional brain abnormalities, as well as the underlying white matter, would be associated with indices of migraine severity and pain catastrophizing.

## Materials and methods

### Participants

Twenty-eight adult volunteers (demographics in Table 1) participated in this study and were compensated for their time. Healthy controls were recruited at the University of Maryland, Baltimore (UMB). Migraine patients were recruited from the Johns Hopkins University (JHU) campuses, local headache clinics, and through community advertisements, and were also enrolled in, but had not yet begun, a larger longitudinal intervention study using intense training in Vipassana Meditation. For the current study, subjects attended 2 sessions, one behavioral testing session and one fMRI scanning session. The two sessions were always on the same day. Migraine patients with high frequency attacks, defined as more headache days than headache-free days in a typical month based on self-report were included in the study. Diagnosis was confirmed by the study physician using the International Classification of Headache Disorders-II (ICHD-II) criteria (Olesen and Steiner, 2004; Olesen, 2008) and data from prospective daily headache diaries that were part of the larger intervention study. All migraine patients had a history of recurring headaches for at least 1 year. Exclusion criteria for all subjects included an unstable psychiatric disorder, pregnancy, illicit drug use, and alcoholism. Healthy controls were additionally excluded for any chronic or current pain, and migraine patients with other pain conditions were excluded. All procedures were approved by the UMB and JHU Institutional Review Boards and in accordance with the 1964 Helsinki declaration and its later amendments or comparable ethical standards, and informed written consent was obtained from each participant prior to any study procedures.

**Table 1:** **Baseline characteristics**.

		**Patients**	**Controls**
N		14	14
Sex	F	11	11
	M	3	3
Age(SD)		40.8(11.9)	38.9(12.5)
Range		18–59	20–61
Highest education	High school	2	1
	Bachelor's degree	7	8
	Graduate degree	5	5
Handedness	R	13	13
	L	1	1
Migraine pain intensity (mean last month, SE)		5.4 (.6)^*^	0
Range		2–10	
Disease duration (years, SE)		10.9 (2.0)^*^	0
Range		1–27	
Migraine frequency (#/last month, SE)		9.6 (2.1)^*^	0
Range		2–30	
Average headache days/28 day month^a^ (from a 3 month daily headache diary)		20.08 (2.0)	-
Range		7.8–28	
Diagnosis	Chronic migraine	10	-
	Episodic migraine	4	-
Medication (past 24 h)	Antidepressant	9	1
	NSAID	5	-
	Triptan	4	-
	Opioid	1	-
	Anticonvulsant	4	-
	Anxiolytic/muscle Relaxant	3	-
	Other prophylactic	5	1
	Other analgesic	1	1
Headache rating before scan		2.1 (.7)^b^	-
Headache rating during scan^c^		3.6 (.8)^d^	-
SFMPQ(SE)	Sensory	4.5 (1.6)^*^	0
	Affective	1.5(.5)^*^	0
	Total	6(2.0)^*^	0
MIDAS(SE)	Total	71.2 (20.3)^*^	.4 (.3)
POMS(SE)	Anger/hostility	12.9 (.5)	12.8 (.4)
	Confusion/bewilderment	13.3 (1.1)^*^	10.7 (.5)
	Tension/anxiety	16.5 (.9)^*^	12.7 (.6)
	Depression/dejection	17.1 (.6)^*^	15.6 (.3)
	Fatigue/inertia	14.4 (1.2)^*^	9.4 (.8)
	Vigor/activity	15.6 (1.4)^*^	23.0 (1.9)
	Total mood disturbance	58.4 (4.1)^*^	38.3 (3.0)
PCS(SE)	Total score	17.7 (2.3)^*^	6.9 (2.1)
Peak stimulus temperature(SE)	P2	47.5°C (0.3°)^†^	46.4°C (0.5°)

* *p < 0.05*,

† *p < 0.10*.

a *Data from prospective daily headache diaries. Migraine pain intensity, frequency, and duration in above rows are from retrospective reports on the day of the scan*.

b *N = 12, pre-scan headache pain ratings were not obtained from 2 patients*.

c *At the end of the scanning session, participants were asked to rate their headache pain during the scan*.

d *N = 13, post-scan headache pain rating was not obtained from 1 patient*.

*SFMPQ, Short-form McGill Pain Questionnaire; MIDAS, Migraine Disability Assessment Scale; POMS, Profile of Mood States; PCS, Pain Catastrophizing Scale*.

### Behavioral session

#### Questionnaires

During the behavioral testing session, all participants completed the Pain Catastrophizing Scale (PCS) (Sullivan et al., 1995), the Profile of Mood States (POMS) (McNair et al., 1971), the short-form McGill Pain Questionnaire (SFMPQ) (Melzack, 1987), the Migraine Disability Assessment Scale (MIDAS), and a demographic questionnaire (Stewart et al., 1999). In addition, migraine patients' self-reported disease duration (years), frequency of migraine attacks in the last month, last 6 months, last year, and last 2 years, and ratings of migraine pain intensity using a 0–10 numerical rating scale (NRS; 0 = no pain and 10 = worst pain imaginable) for the last 24 h, last week, and last month. Patients were also asked to rate their current migraine pain intensity using the NRS before and after each scan session (Table 1).

#### Quantitative sensory testing (QST)

All subjects underwent two psychophysical protocols and a practice session of the fMRI protocol. In all protocols, thermal heat stimuli were delivered to the volar forearm with a contact probe (30 × 30 mm Medoc Pathway ATS Peltier device; Medoc Advanced Medical Systems Ltd., Ramat Yishai, Israel). In the first protocol, we used a levels procedure to determine the rate of change in pain intensity and unpleasantness with increasing temperature. In this procedure, subjects received a series of 6 s stimuli delivered with an ascending order of target temperature (35, 37, 39, 41, 43, 45, 47, and 49°C) from a baseline temperature of 32°C with a ramp rate of 4.4°C/s. Therefore, the time to peak stimulus varied based on the target temperature. Each stimulus was flanked with a 6 s baseline (32°C) both before and after each sequence. After the stimulus for each target temperature, subjects provided a rating of pain intensity, and pain unpleasantness on a numerical rating scale. If subjects reported a 10/10 prior to the highest temperature, the procedure was aborted at that temperature.

Next, we performed a ratings procedure where subjects received 6 s stimuli with variable ramp rates (4.4–10.0°C/s) for different temperatures, to ensure that the ramp time to reach the target temperature was matched (1.6 s). The target temperatures (39, 40, 41, 42, 43, 44, 45, 47, 48, 49°C) were delivered pseudorandomly, with each stimulus presented 1–3 times. After each stimulus, subjects would provide a rating of pain intensity and pain unpleasantness on a numerical rating scale. Importantly, in both protocols, the thermode was moved after each stimulus to avoid temporal summation.

#### Pre-scan practice session

The fMRI stimuli were determined during the behavioral session. Thermal stimuli were delivered using the same contact probe (30 × 30 mm) for 8–12 s (rectangular distribution) with a ramp time of 1.6 s from baseline to peak temperature (i.e., ramp rate varied based on the target peak temperature). The stimuli were applied to the left volar forearm, separated by intervals (4–8 s, rectangular distribution) of baseline temperature (32°C). P2—the peak temperature used in both the practice session and the fMRI protocol—was determined at the beginning of the pre-scan session, using a simple ramp-and-hold procedure, at the temperature at which the participant rated pain intensity about 5–6 on a 0 (no pain) to 10 (most intense pain imaginable) numerical rating scale. P1 was set at 1°C below P2, and P0 was set at 37°C for all participants. Subjects practiced the fMRI protocol—performing a cognitive task (see details below) while receiving nociceptive stimulation. After performing the cognitive task, subjects provided pain intensity and unpleasantness ratings using a computer key-pad and recorded using E-Prime 2.0 software (Psychology Software Tools, http://www.pstnet.com).

### Scanning session

#### fMRI stimuli

The tailored stimuli described above were delivered in the scanner by an fMRI-compatible probe (30 × 30 mm). Participants did not provide pain ratings during the scanning session. The scan session included two fMRI runs (Run 1: 9 min 20 s, Run 2: and 9 min 32.5 s) consisting of nine stimuli each at three different temperatures [no pain (P0), mild pain (P1), and moderate pain (P2)] for a total of 27 thermal stimuli per run. The same temperature was used throughout the pre-scan and scanning sessions for all subjects, with the exception of one patient who requested that P2 be lowered by 1°C in for the second functional run.

Subjects experienced pain while performing a modified Attentional Network Test (ANT) (Fan et al., 2002). Briefly, subjects were instructed to identify the direction of a central arrow, while ignoring the direction of flanking arrows. There were two levels of task difficulty: easy and difficult. In the easy task, the flanking arrows were congruent to the central arrow, whereas in the difficult task the flanking arrows were incongruent. There were no group differences in task performance (reaction time or accuracy; see: Mathur et al., 2015). The current study focuses on the main effect of pain. Performing the task did not affect pain intensity *F*_(1, 26)_ = 0.5, *p* = 0.49 or unpleasantness *F*_(1, 26)_ = 1.6, *p* = 0.21 (three-way repeated measures ANOVA including group, task condition, and pain condition). We previously reported that patients had altered brain responses to pain-cognition interactions (Mathur et al., 2015). During concurrent pain and task performance, patients had decreased task-related activity but increased task-related reductions in pain-related activations compared to controls.

#### MRI scanning

The MRI session included the following scans: anatomical, functional resting-state, two functional pain and task runs, and diffusion weighted (DWI) (for gray matter and resting-state results see: Hubbard et al., 2014). Images were acquired in a single session using a Siemens 3T Tim Trio MRI scanner equipped with a 12-channel head coil. Total scan time was approximately 50 min. Scan parameters included: anatomical: high-resolution T1-weighted MPRAGE [144 slices, repetition time (TR) 2500 ms, echo time (TE) 3.44 ms, flip angle 9.0°, FOV 230 mm, resolution 0.9 × 0.9 mm, matrix size 256 × 256 mm, slice thickness 1 mm, no gap]; functional whole-brain images: T2^*^-weighted, echo planar imaging sequences (spin-echo, 36 slices, TR 2500 ms; TE 30 ms; flip angle 90°; FOV 230 mm, resolution 1.8 × 1.8, matrix size 128 × 128 mm, slice thickness 4 mm, no gap, oblique slices); DWI: 64 directions, 5 B0 images (spin-echo, 72 slices, TR 9000 ms; resolution 1.8 × 1.8, matrix size 128 × 128 mm, slice thickness 2 mm, axial slices).

### Analyses

#### QST analyses

Repeated-measures ANOVA was used to test a group-by-temperature interaction on pain intensity and unpleasantness ratings.

#### Pre-scan practice session analyses

For the pre-scan practice of the fMRI task, repeated-measures ANOVA was used to test the main effects of “group” (two-levels: “patients,” “controls”) and “temperature” (three levels: “P0,” “P1,” and “P2”), and their interaction on pain intensity and unpleasantness ratings. Significant results were further explored by examining the simple effects using *t*-tests. Additionally, if results revealed a group difference in stimulus temperature (P2), individual stimulus temperature would be entered into the relevant models as a covariate to ensure group effects were not due to differences in individualized stimulus temperature.

#### fMRI analyses

Functional brain images were preprocessed and analyzed using SPM8 software (Wellcome Trust Centre for Neuroimaging, UCL, London, UK) implemented in Matlab (v8.0.0.783, Mathworks, Nantick, MA). Preprocessing included slice timing correction, motion correction, coregistration of the anatomical image to the mean functional volume, segmentation into three different tissue classes (CSF, white matter, and gray matter), normalization to the Montreal Neurological Institute (MNI) template, spatial smoothing at 8 mm FWHM.

A general linear model was defined for each participant, which included six regressors of interest that investigated the various task and pain level combinations: hard/P2, hard/P1, hard/P0, easy/P2, easy/P1, easy/P0. All trials included both a thermal stimulus and a cognitive task. In order to model only the period where cognitive load and thermal stimulation were stable and concurrent within each trial, the first and last portions of each trial were excluded, as follows: the first 1.4 s of each task trial corresponded to a slight overshoot in thermal stimulation, and the last 1.4 s included the beginning of the down-ramp. Excluding the first and last 1.4 s of the task resulted in modeled trials ranging 5.7–11.8 s in length that included both a stable heat stimulus and a concurrent task. The six motion parameters were included as covariates of no interest, and reaction time on the task was entered as a parametric modulator to control for task difficulty. No other regressors were included.

Pain-related brain activations were investigated as follows. First, a pain vs. no pain contrast [(P1+P2) vs. P0] was tested. Brain activations common to both groups (patients and controls) were identified with a conjunction analysis testing the conjunction null hypothesis, as implemented in SPM (Friston et al., 2005), and group differences of whole brain pain-related activations were tested with an independent-samples *t*-test. Next the two levels of nociceptive stimulation (P1 vs. P2) were compared. First, a conjunction analysis was performed to identify common regions of difference between P1 and P2 across patients and controls. Next, an independent-samples *t*-test was used to test for group differences in P1 and P2. Because there was a group difference in pain intensity evoked by P2 during the pre-scan practice session, pre-scan pain intensity ratings were included as a covariate to ensure significant group differences in brain activations were not due to differences in pain intensity.

### Correlations with PCS and disease severity

Given the relationship between PCS and migraine symptomatology, here we aimed to test the relationship between of PCS and acute pain processing. We also investigated the relationship and indices of disease severity (disease duration, migraine frequency in the last month, and average intensity in the last month) and acute pain processing. To do so, contrast images (e.g., [(P1+P2) vs. P0]) were created at the individual subject level, and these whole-brain contrast images were correlated with individual ratings (PCS and indices of disease severity).

For all fMRI analyses, an initial voxelwise threshold of *p* < 0.005 and cluster size >25 voxels was used, corrected for multiple comparisons at the cluster level (*p* < 0.05), unless otherwise stated. More stringent thresholds were used to separate overlapping clusters when necessary for subsequent extraction.

To visualize individual differences in brain activity depicted in the figures, we used MarsBar (Brett et al., 2002) to extract beta values from regions of interest (ROIs), defined as significant clusters identified by planned contrasts.

#### DTI analyses

Diffusion weighted images were preprocessed with the TORTOISE software package (Pierpaoli et al., 2010). Data were imported, and B-matrix gradients were computed, motion, eddy-current and EPI distortion corrected, and then brain extracted (Rohde et al., 2004; Wu et al., 2008). A tensor model was then fit, and fractional anisotropy (FA) images were then produced for voxelwise statistical analysis in the FMRIB Software Library (FSL v.5.1, Oxford, UK; Smith et al., 2004) tract-based spatial statistics toolbox (TBSS; Smith et al., 2006). The subjects' FA maps were aligned to a common template using non-linear registration. Next, a mean FA image was calculated and thinned to a single voxel in width to create a mean FA skeleton, representing the center of all white matter tracts common to all subjects. Each subject's aligned FA values were projected onto the skeleton. Non-parametric, voxelwise statistics were performed using the randomize toolbox in FSL (Winkler et al., 2014). Two skeletons were created—one for the patients and one for the controls, in order to perform regression analyses on the patients, and to extract mean FA values of the significant regions in controls. All analyses in DTI were defined *a priori* to probe relationships identified between the pain-related neural responses in migraine patients and disease severity variables. We investigated the correlations between FA and (1) disease duration, (2) migraine frequency in the last month, (3) migraine intensity in the last month, and (4) PCS. Significance was determined using non-parametric permutation testing (Monte Carlo Simulation) of the mean TBSS skeleton in AlphaSim, as part of the AFNI software package (version2011_12_21_1014, afni.nimh.nih.gov/). The corrected alpha value of < 0.01 was achieved with a combination of a *p* < 0.005 and a cluster extent of 4 voxels.

## Results

### Behavioral results

Demographic and behavioral data are summarized in Table 1.

#### QST results

**Figure 1:**
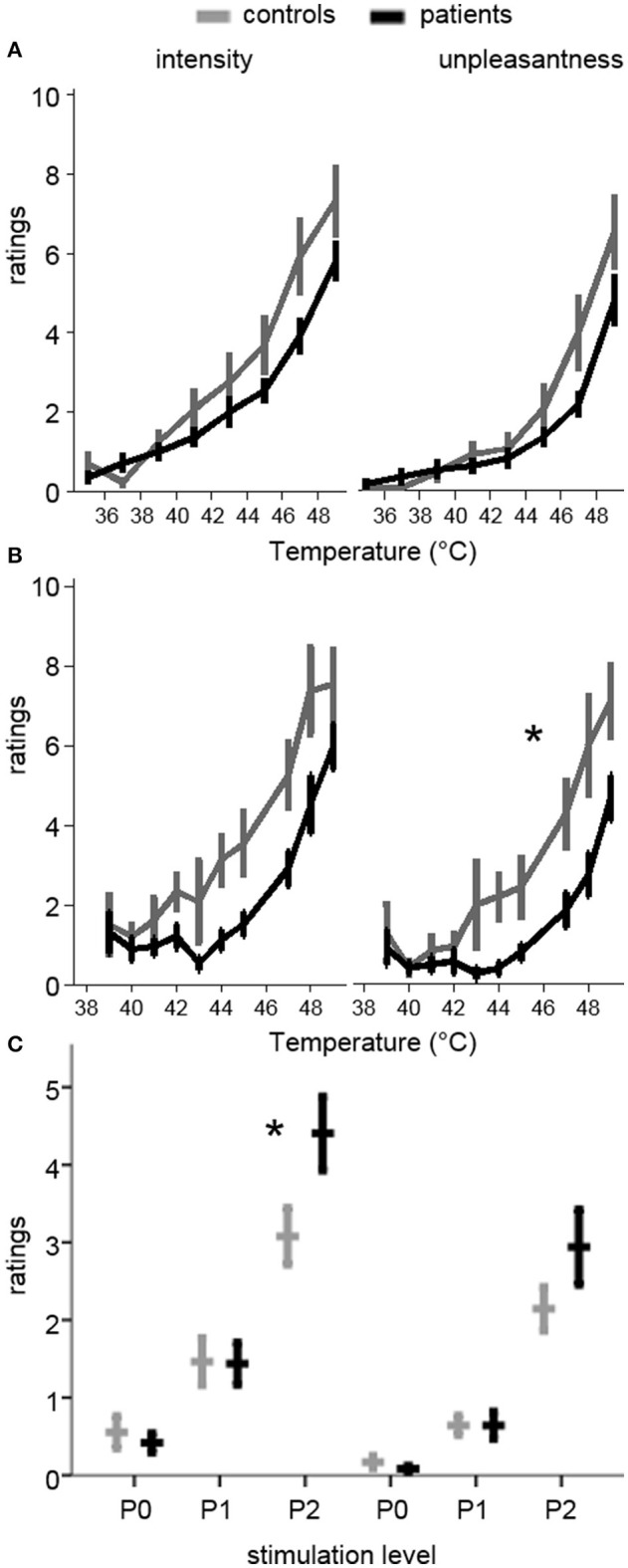
**Pain ratings during quantitative sensory testing and pre-scan ratings of the stimulation levels used in the MRI session**. Ratings are on a 0–10 scale. Plots in the left column are for pain intensity, while those on the right are for pain unpleasantness. **(A)** Ratings during the Ratings test (increasing temperatures from 35 to 49°C). Patients had a non-significant trend toward lower ratings (rightward shift of the curve). **(B)** Ratings during the Levels test (pseudo-randomly delivered temperatures between 39 and 49°C). Patients had significantly lower unpleasantness ratings (rightward shift), and a trend toward lower intensity ratings. **(C)** Ratings for the temperatures used in the pre-scan session, where P0 was a non-painful temperature, P1 was mildly painful, and P2 was moderately painful, based on the ratings during Levels and Ratings. The same temperatures for each subject were used in the MRI session. Patients had significantly higher intensity ratings for P2. ^*^p < 0.05 in **(B)** RM-ANOVA for group-by-temperature interaction and in **(C)** significant group-by-level interaction and simple effect, controlling for temperature.

Ratings and levels data were available for 11 patients and 13 controls. Patients had a significant rightward shift in pain unpleasantness [*F*_(9, 198)_ = 2.4, *p* = 0.015, $\eta_{p}^{2}=$ 0.10], but not for intensity [*F*_(9, 198)_ = 1.3, *p* = 0.247, $\eta_{p}^{2}=$ 0.06] during the ratings protocol. In the levels protocol, there was a non-significant trend for both unpleasantness [*F*_(7, 154)_ = 2.0, *p* = 0.055, $\eta_{p}^{2}=$ 0.08] and intensity [*F*_(7, 154)_ = 1.9, *p* = 0.070, $\eta_{p}^{2}=$ 0.08], suggesting that patients were less sensitive to thermal stimuli (Figure 1).

#### Pre-scan practice session results

All 28 participants completed the practice session outside of the scanner. Individualized peak thermal stimulus used in the practice and fMRI sessions was marginally lower in controls compared to patients [P2 temperature (S.E.M.): Patients 47.4°C (0.3); Controls 46.4°C (0.5); *t*_(26)_ = 2.0, *p* = 0.06, *d* = 0.73], suggesting lower perceptual sensitivity among patients. P1 was set at 1°C below P2, and P0 was set at 37°C. There were no significant main effects of group on pain intensity or unpleasantness ratings, although there was a significant group-by-pain interaction [*F*_(2, 52)_ = 6.1, *p* = 0.004, $\eta_{p}^{2}=$ 0.19], such that patients reported greater pain intensity than controls during P2 [Patients 4.4 (0.33); Control 3.1 (0.28); *t*_(26)_ = 2.2, *p* = 0.04, *d* = 0.83]. This effect remained after controlling for stimulus temperature [*F*_(2, 50)_ = 4.55, *p* = 0.02, $\eta_{p}^{2}$ = 0.15]. The corresponding interaction on pain unpleasantness ratings was not significant [Patients 2.9 (0.34); Control 2.1 (0.22); *F*_(2, 52)_ = 2.5, *p* = 0.09, $\eta_{p}^{2}=$ 0.09]. The difference in pain ratings between P2 and P1 was also significantly greater in patients than controls for intensity [*t*_(26)_ = 2.9, *p* = 0.008, *d* = 1.09], but not unpleasantness [*t*_(26)_ = 1.8, *p* = 0.09, *d* = 0.66], even after controlling for peak temperature [*F*_(1, 25)_ = 6.3, *p* = 0.02, $\eta_{p}^{2}=$ 0.20]. Ratings did not differ for P1 between patients and controls for intensity [Patients 1.4 (0.20); Control 1.5 (0.22)] or unpleasantness [Patients 0.64 (0.14); Control 0.64 (0.10)] (Figure 1).

#### Behavioral results summary

In summary, patients were less sensitive to noxious thermal stimulation than controls. Higher temperatures were selected for patients to account for this difference, but pain ratings were higher for patients than controls at P2. To ensure that this did not drive any group-level differences in pain-related activations, fMRI models that showed significant group effects were also run controlling for group differences in pre-scan pain intensity ratings.

### fMRI results

#### Pain-related neural activity

**Figure 2:**
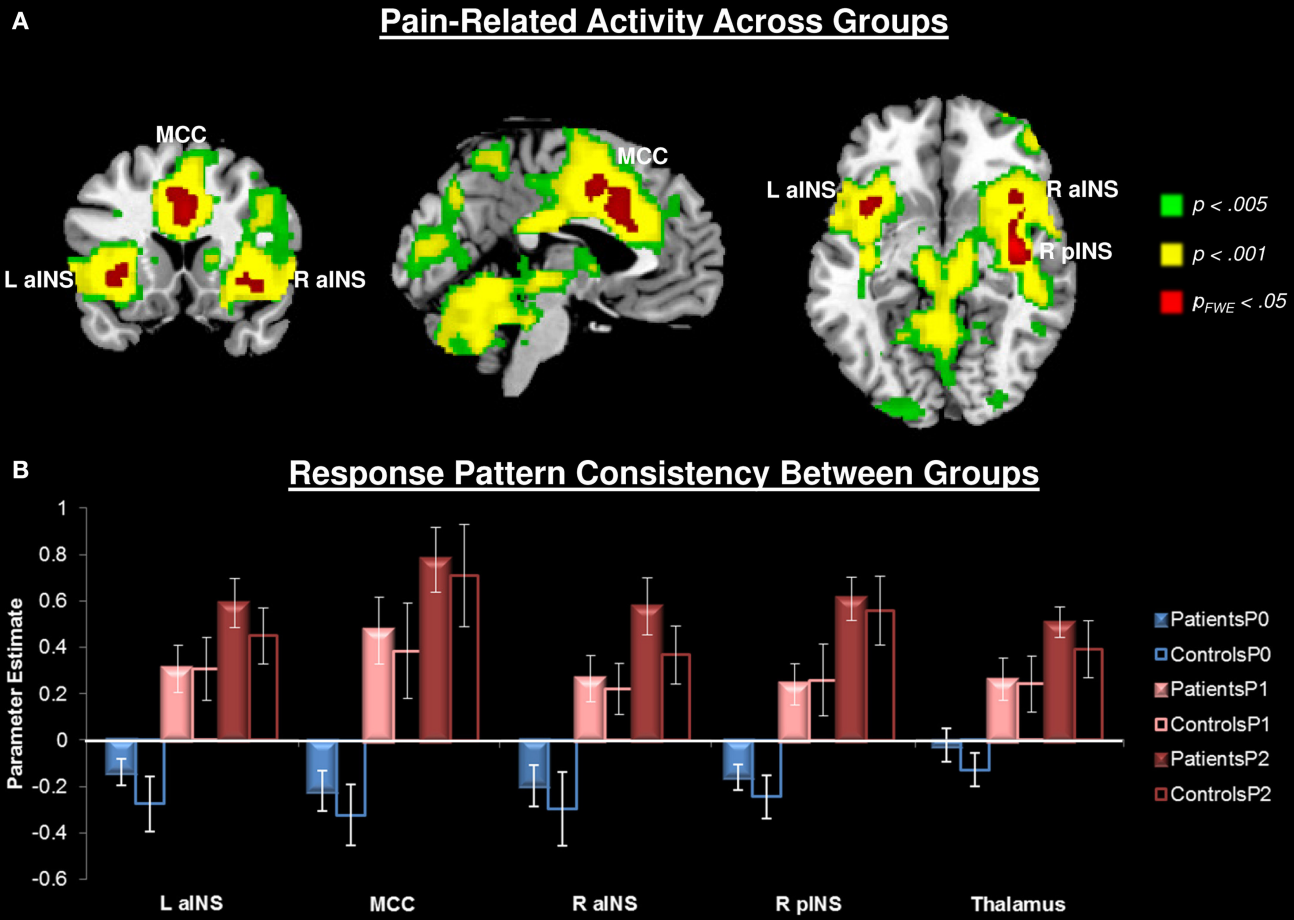
**Pain-related neural responses among all participants (*n* = 28)**. Pain was evoked with a contact heat thermode applied to the participants' left volar forearm. **(A)** Whole brain contrast [Pain (P1+P2) > No Pain (P0)], at the a priori defined threshold *p*_*uncorrected*_ < 0.005 (green), a more stringent threshold of *p*_*uncorrected*_ < 0.001 (yellow), and the most conservative *p*_*fwe*_ < 0.05 (red). For subsequent analyses, activity was extracted from the most conservative threshold (red clusters) to isolate separable clusters. Right thalamus also activated at this threshold, but is not seen at these slices. **(B)** Extracted activity from significant (*p*_*fwe*_ < 0.05) clusters: left anterior insula (L aINS), midcingulate cortex (MCC), right anterior insula (R aINS), right posterior insula (R pINS), and thalamus demonstrates that response pattern was consistent across groups. No pain (P0), mild pain (P1), moderate pain (P2).

Painful heat stimuli were associated with neural activity within regions known to be associated with nociceptive processing (Figure 2, Table 2). In order to separate overlapping suprathreshold clusters, we used a stringent voxelwise threshold (*p* < 0.05, family-wise error corrected). Five clusters survived this threshold: P1 and P2 were associated with activity in the mid-cingulate cortex (MCC), bilateral anterior INS (aINS), right (contralateral to the stimulus) posterior INS (pINS), and right thalamus. To confirm that activation in each of these clusters was significant within each group, we performed paired *t*-tests on the mean signal for P1 + P2 > P0. We found that in all cases the contrast was significant for each cluster for each group (*p* < 0.002). The right primary somatosensory cortex (S1) was also activated, but did not survive the cluster-level threshold (*p* = 0.07). No significant pain-related areas of deactivation survived the cluster-level correction.

**Table 2:** **Pain-related neural activity**.

**Contrast**	**Region**	**Peak *T-value***	**Cluster extent**	**Peak MNI coordinates**		
				***X***	***Y***	***Z***
**ALL PARTICIPANTS**						
[Pain (P1 + P2) > No Pain (P0)]; ^*^Adjusted threshold: *p_fwe_*< 0.05, *k* > 25						
	R pINS (ext. to mINS)	9.75	896	40	−10	−8
	MCC	7.46	879	6	4	48
	L aINS/mINS	7.01	275	−36	2	0
	R thalamus	6.52	35	14	−12	−2
	R aINS	6.27	42	40	16	−8
	*R S1*	*6.04*	*28*	*24*	−*40*	*60*
**ALL PARTICIPANTS**						
[Moderate Pain (P2) > Mild Pain (P1)]; ^*^Adjusted threshold: *p* < 0.001, *k* >25						
	R pINS (ext. to mINS)	6.78	3340	40	−18	20
	L cerebellum	6.57	716	−24	−42	−32
	aMCC	5.78	198	4	24	18
	L lateral PCC	5.43	463	−16	−30	44
	R S1	5.28	1220	22	−32	58
	L IPL	4.97	235	−56	−32	22
	L precuneus	4.55	307	−12	−66	32
	R PHG	4.19	87	16	−12	−8
	R cuneus	4.17	122	8	−84	12
**PATIENTS > CONTROLS**						
**[Moderate Pain (P2) > Mild Pain (P1)]**						
	*R mINS*	*4.40*	*42*	*30*	*2*	*14*
**CONTROLS > PATIENTS**						
**[Moderate Pain (P2) > Mild Pain (P1)]**						
	L SPL ext. to STG	4.75	1139	−36	−66	46
	L MTG	4.66	277	−60	−38	−2
	L DLPFC	4.41	431	−54	28	18
	*dorsal aMCC*	*3.59*	*95*	*2*	*30*	*48*

p < 0.005, cluster-level correction p < 0.05 for all contrasts unless otherwise noted in left column(^*^). Cluster extent is in voxels. Italicized regions did not survive cluster level threshold but were included based on a priori hypotheses. pINS, Posterior insula; mINS, middle insula; MCC, midcingulate cortex; aINS, anterior insula; S1, primary somatosensory cortex; aMCC, anterior midcingulate cortex; PCC, posterior cingulate cortex; IPL, inferior parietal lobule; PHG, parahippocampal gyrus; SPL, superior parietal lobule; STG, superior temporal gyrus; MTG, middle temporal gyrus; DLPFC, dorsolateral prefrontal cortex.

#### Modulation of pain-related activity by stimulus intensity

Both pain stimuli (P1 and P2) were associated with similar patterns of activity across all participants (Figure 2). To compare brain activity related to the two pain stimuli, we used a slightly more stringent threshold (*p* < 0.001, cluster-level correction *p* < 0.05). Compared to P1, P2 was associated with greater activity within regions commonly associated with nociceptive processing (Figure 2, Table 2). There were no regions that showed significantly greater activity to P1 compared to P2.

#### Group differences in pain-related activity

**Figure 3:**
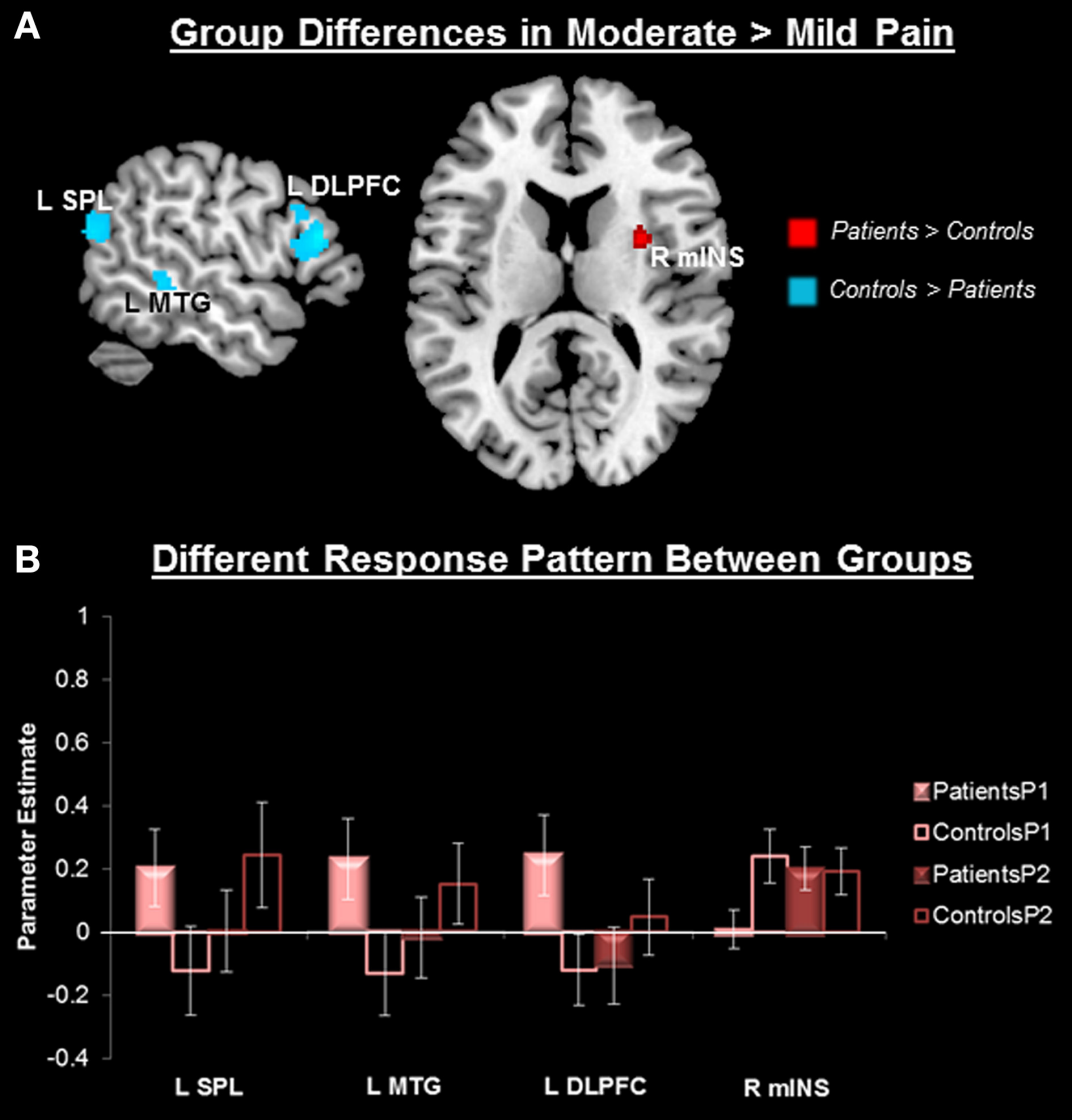
**Group differences in pain-related neural response. (A)** Whole-brain independent sample *t*-test conducted on the [Moderate Pain (P2) > Mild Pain (P1)] contrast, *p* < 0.005, cluster-level correction *p* < 0.05. Insula cluster displayed at *p*_*uncorrected*_ < 0.005. Red, Patients > Controls; Blue, Controls > Patients. **(B)** Extracted activity from the left superior parietal lobule extending to the superior temporal gyrus (R SPL), left middle temporal gyrus (L MTG), left dorsolateral prefrontal cortex (L DLPFC), and right middle insula (R mINS), demonstrates that the pattern of response to mild and moderate pain differed between patients and controls. Mild pain (P1), moderate pain (P2).

No group differences in overall pain-related (P1 + P2 > P0) neural activity survived our original threshold (*p* < 0.005, *k* > 25, uncorrected) nor a liberal threshold (*p* < 0.05, uncorrected). Compared to controls, patients showed greater activity in response to P2 compared to P1 in the right middle INS (mINS) (at *p* < 0.005, uncorrected, *k* > 25), but this did not survive cluster-level correction (Figure 3, Table 2). Controls showed greater activity than patients in this contrast (P2 > P1) in the left superior parietal lobule (SPL), the left superior temporal gyrus (STG), left middle temporal gyrus (MTG), left DLPFC, and dorsal anterior MCC (aMCC) (Figure 3, Table 2). All group-pain interactions remained after controlling for group differences in pre-scan pain intensity ratings.

#### Pain catastrophizing and pain-related activity in migraine

**Figure 4:**
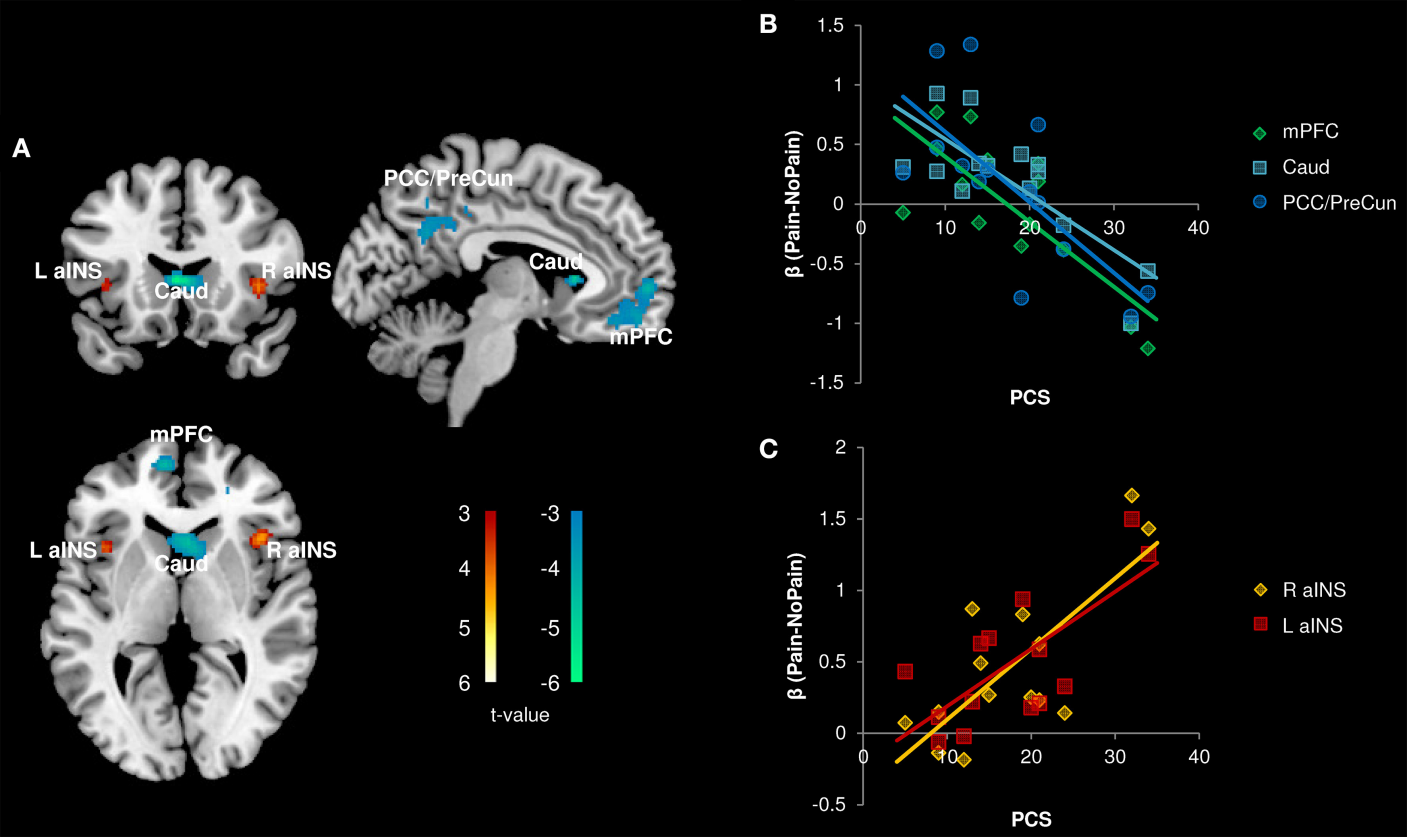
**Correlation with Pain Catastrophizing Scale (PCS) scores (*n* = 14)**. **(A)** Whole brain correlations conducted on the [Pain (P1+P2) > No Pain (P0)] contrast, *p* < 0.005, cluster-level correction *p* < 0.05. Insula clusters displayed at *p*_*uncorrected*_ < 0.005. **(B)** Extracted activity from caudate (Caud), medial prefrontal cortex (mPFC), and posterior cingulate cortex/precuneus (PCC/PreCun) clusters plotted against PCS scores. **(C)** Extracted activity from left anterior insula (L aINS) and right anterior insula (R aINS) clusters plotted against PCS scores. Warm colors, positive correlation; Cool colors, negative correlation.

In the patient group, we performed a whole brain correlation between functional brain activity and PCS. We found that PCS was negatively correlated with activity in the medial prefrontal cortex (mPFC), caudate, and posterior cingulate cortex (PCC)/precuneus (Figure 4, Table 3). Bilateral aINS were also positively correlated with PCS among patients.

**Table 3:** **Correlation between pain-related activity and clinical measures in patients**.

**Clinical index**	**Region**	**Cluster size**	**Peak *T*-value**	**Peak MNI coordinates**		
				**X**	**Y**	**Z**
**PAIN CATASTROPHIZING**						
**Positive correlation**						
	*R aINS*	*78*	*4.36*	*42*	*22*	*6*
	*L aINS*	*25*	*3.79*	−*40*	*16*	*4*
**Negative correlation**						
	Caudate	229	6.02	−4	20	6
	mPFC	979	5.97	8	46	−14
	PCC/precuneus	451	4.09	−10	−56	32
**MIGRAINE PAIN INTENSITY**						
**Positive correlation**						
	*R pINS*	*56*	*4.77*	*42*	−*14*	−*4*
	*L aINS*	*69*	*4.48*	−*42*	*12*	*6*
	*R aINS*	*57*	*3.73*	*44*	*18*	*0*
**Negative correlation**						
	PCC	745	5.81	−4	−48	12
	mPFC	1307	5.77	−8	44	−2
**MIGRAINE FREQUENCY**						
**Positive correlation**						
	*L pINS*	*48*	*4.43*	−*46*	−*16*	*20*
	*R pINS*	*75*	*4.42*	*36*	−*26*	*20*
**DISEASE DURATION**						
**Positive correlation**						
	*L STG*	*294*	*5.04*	−*56*	−*4*	*4*

*p < 0.005, cluster-level correction p < 0.05 for all contrasts. Cluster size is in voxels. Italicized ROIs did not survive cluster level correction but were included based on a priori hypotheses. mPFC, Medial prefrontal cortex; PCC, posterior cingulate cortex; aINS/pINS, anterior/posterior insula; STG, superior temporal gyrus. The contrast in all cases was [Pain (P1+P2) > No Pain (P0)]*.

#### Disease severity and pain-related activity in migraine

**Figure 5:**
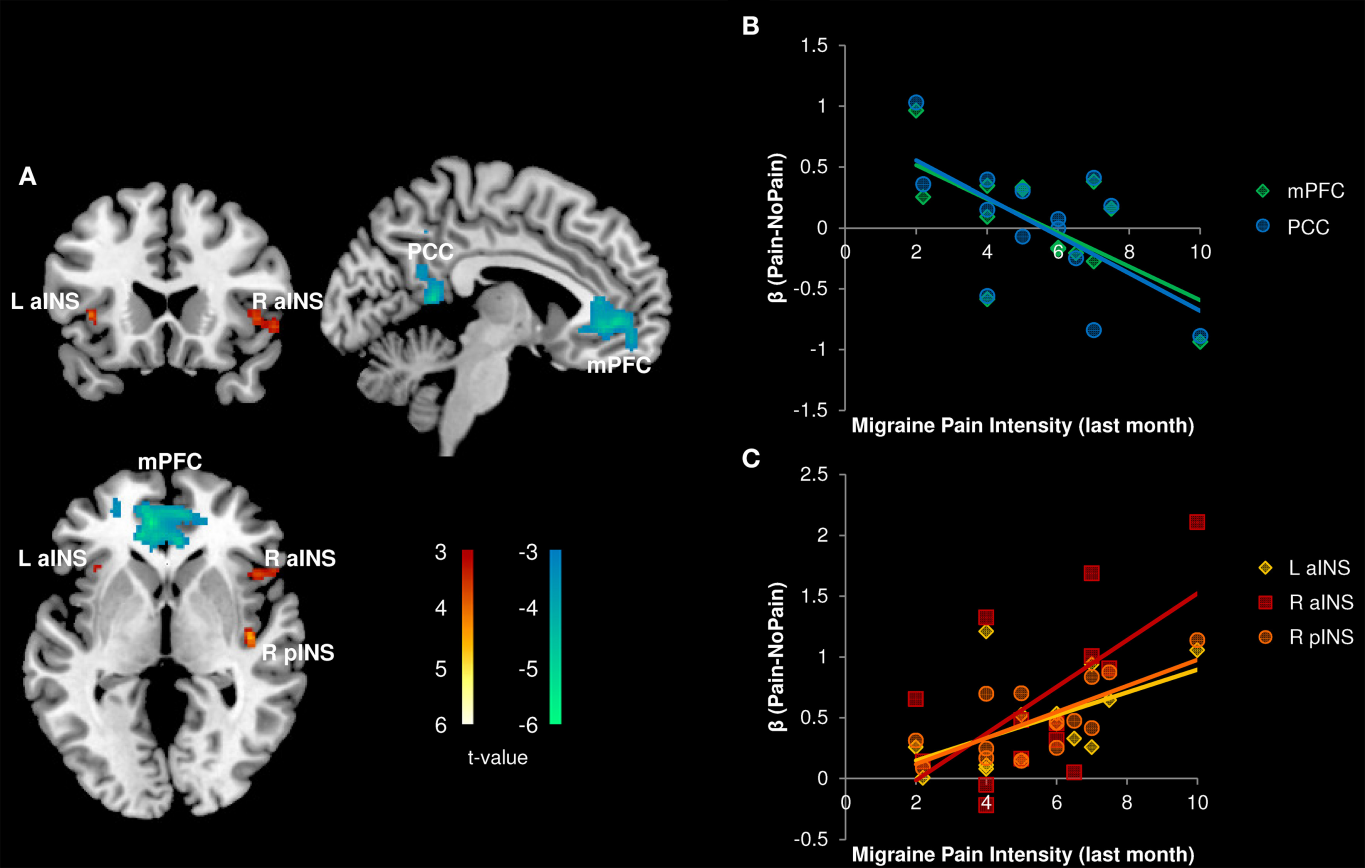
**Correlation with Migraine Pain Intensity (*n* = 14)**. **(A)** Whole brain correlations conducted on the [Pain (P1+P2) > No Pain (P0)] contrast, *p* < 0.005, cluster-level correction *p* < 0.05. Insula clusters displayed at *p*_*uncorrected*_ < 0.005. **(B)** Extracted activity from medial prefrontal cortex (mPFC), and posterior cingulate cortex (PCC) clusters plotted against migraine pain intensity ratings for the last month. **(C)** Extracted activity from left anterior insula (L aINS), right anterior insula (R aINS), and right posterior insula (R pINS) clusters plotted against migraine pain intensity ratings for the last month. Warm colors, positive correlation; Cool colors, negative correlation.

**Figure 6:**
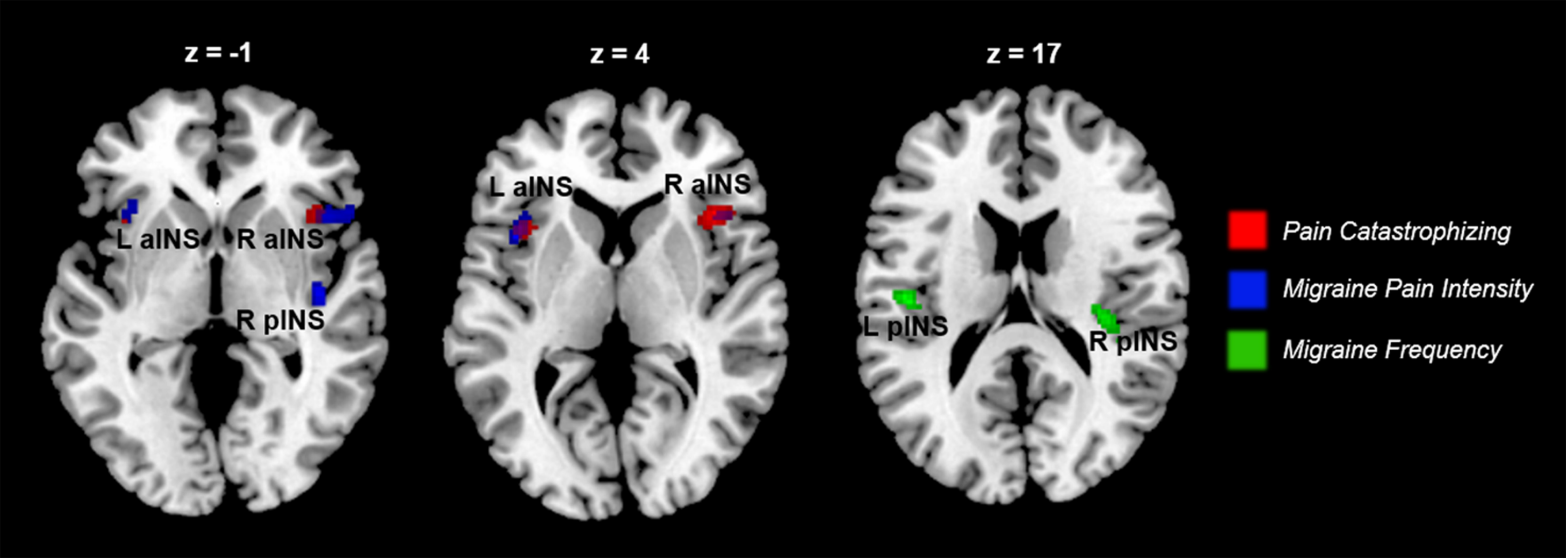
**Indices of disease severity are associated with differential patterns of pain-related insula activations (*n* = 14)**. Whole brain correlations conducted on the [Pain (P1+P2) > No Pain (P0)] contrast, using pain catastrophizing (Red), migraine pain intensity (Blue), and migraine frequency (Green) as covariates of interest. Purple areas show the overlap between catastrophizing and migraine pain-related clusters. Clusters identified at *p*_*uncorrected*_ < 0.005, but displayed at *p*_*uncorrected*_ < 0.01 for better visualization of cluster independence and overlap.

All correlations between pain-related activity and indices of disease severity can be found in Table 3. Migraine frequency and pain intensity over the last month, disease duration, and catastrophizing were not correlated to each other (all *p* > 0.10). Migraine pain intensity in the last month was negatively associated with mPFC and PCC pain-related activity and positively associated with pain-related activity within the bilateral aINS and right pINS (Figure 5, Table 3). Migraine frequency in the last month was positively correlated with activity in the bilateral pINS (Figure 6, Table 3). Disease duration was negatively correlated with activity in the left STG.

#### Disease severity and white matter structure in migraine

Several clinical measures (PCS, migraine pain intensity and frequency in the last month) were found to be associated with increased pain-related activity in the insula (Figures 4, 5). In a *post-hoc* analysis, we investigated whether these indices of disease severity were associated with white matter FA. Whole brain correlations with clinical measures can be found in Table 4. We identified two significant clusters in the right mid-insula where FA and migraine duration were negatively correlated and a significant positive correlation between FA in the left mid insula and PCS (Table 4).

**Table 4:** **Correlation between FA and clinical measures in patients**.

**Clinical characteristic**	**White matter tract**	**Nearest gray matter**	**Cluster extent (voxels)**	**Peak MNI coordinates**		
**Duration**				**X**	**Y**	**Z**
**Negative correlation**						
	Superior corona radiata	R DLPFC	28	40	10	44
	Forceps major/optic radiation	L V1	21	−13	−95	7
	Anterior thalamic radiation/uncinate fasciculus	L lateral frontal pole	19	−30	47	1
		R Cerebellum—Crus II	17	14	−65	−42
	Superior corona radiata	R DMPFC/pre-SMA	14	11	24	53
	Anterior thalamic radiation/uncinate fasciculus	L VLPFC/Lat OFC	12	−36	36	−3
	Inferior fronto-occipital fasciculus/uncinate fasciculus	L Lat OFC	12	−30	27	−11
	Inferior longitudinal fasciculus	R middle temporal gyrus	12	57	−12	−18
	Cingulum/superior corona radiata	L paracentral lobule	11	−15	−55	52
	Forceps major/optic radiation	L occipital pole	10	−24	−91	4
	Superior corona radiata	L DMPFC/pre-SMA	9	−8	26	53
	Fornix	L mediodorsal thalamus	8	−4	−3	4
	Forceps major/optic radiation	R V1	6	11	−86	17
	Anterior thalamic radiation	R VLPFC	6	31	44	16
	Forceps major/optic radiation	L occipital pole	6	−22	−90	10
	External/extreme capsules	R mid insula	6	35	−1	−2
	External/extreme capsules	R mid insula	6	36	−6	−5
		R cerebellum—Crus II	6	23	−74	−39
	Superior corona radiata	L superior parietal lobule	5	−14	−53	57
	Forceps major/optic radiation	L occipital pole	5	−22	−88	13
	Superior corona radiata	L premotor cortex	5	−45	15	13
	Anterior thalamic radiaton/forceps minor	R frontal pole	5	15	54	−7
	Corticobulbar tract	R midbrain	5	10	−7	−11
	Stria terminalis/unicinate Fasciculus	R anterior hippocampus	5	32	−15	−29
	Stria terminalis/unicinate Fasciculus	R anterior hippocampus/parahippocampal cortex	5	28	−4	−34
	Superior corona radiata	R DMPFC/pre-SMA	4	−8	22	56
	Superior longitudinal fasciculus/superior corona radiata	L middle frontal gyrus	4	−25	20	31
	Anterior thalamic radiation	L VLPFC	4	−38	32	8
	Fornix	R thalamus	4	2	0	6
	Superior longitudinal fasciculus (temporal)/Inferior longitudinal fasciculus	L inferior temporal gyrus	4	−49	−58	−5
	Anterior thalamic radiation/Forceps minor	R frontal pole	4	27	49	−7
		R cerebellum—Crus II	4	18	−66	−37
		R cerebellum—IX	4	11	−53	−47
**MIGRAINE FREQUENCY**						
**Positive correlation**						
	Superior corona radiata	R S1/M1	11	26	−20	44
**Negative correlation**						
	Superior corona radiata	R DMPFC/SMA	9	5	5	52
**PCS**						
**Positive correlation**						
	Superior corona radiata	R DLPFC	21	18	4	60
	External/extreme capsules	L mid insula	6	−34	13	−5
	Superior corona radiata	R DLPFC	5	19	5	58
	Anterior thalamic radiation	R VLPFC	4	−34	38	−3
**PAIN INTENSITY**						
**Negative correlation**						
	Superior longitudinal fasciculus/superior corona radiata	R DLPFC	4	−36	20	26

## Discussion

Migraine is a prevalent, debilitating pain disorder associated with central structural and functional abnormalities. Specifically, low-frequency migraine is associated with aberrant trigeminal nociceptive processing, sometimes extending to extra-trigeminal regions—however there is no direct evidence of such abnormalities among patients with frequent migraine attacks. Here, we examined whether high-frequency migraine patients had abnormal pain-related brain responses to nociceptive stimuli outside the trigeminal system. We report two main findings. First, migraine patients had reduced activity in pain modulatory brain regions, and enhanced activity in a nociceptive processing region, in response to moderate vs. mild pain. Second, evoked pain-related activity and white matter structure of the insula correlated with pain catastrophizing and indices of migraine severity.

In addition, we provide evidence that high frequency migraine patients have aberrant suprathreshold nociceptive processing in an extra-trigeminal body area. Pre-scan pain QST revealed an unexpected rightward shift in the nociceptive stimulus-response curve for pain unpleasantness. Specifically, although pain thresholds on the volar forearm were similar between migraine patients and controls, at higher temperatures patients reported lower pain intensity and unpleasantness compared to controls. Patients also required a higher temperature in the scanning session to obtain a moderate pain level (defined by a 6/10 on a numerical rating scale), further supporting this interaction between group and stimulus intensity on pain ratings. These findings suggest that migraine patients were less sensitive to noxious thermal stimulation of the arm. These findings are corroborated by a previous study of extratrigeminal thresholds in patients with high frequency migraine (de Tommaso et al., 2003).

Significant group by stimulus intensity interactions were also found in pain-related brain response. Specifically, migraine patients did not activate pain modulation and cognitive control regions, including the left DLPFC, left SPL/STG, and left MTG, during moderate pain, but did do so during low pain. In contrast, controls showed significant activations only at moderate pain levels (Figure 3). A similar result was previously observed in migraine patients with nociceptive stimulation to the face, where the DLPFC was activated at a lower temperature, but not at high temperatures, and controls only showed activation at the higher temperature (Russo et al., 2012b). Several lines of evidence suggest that a pain modulatory role for the DLPFC. First, activity in this region negatively correlates with percepts of intensity and unpleasantness to an experimental acute pain stimulus (Lorenz et al., 2003), and chronic pain patients have been shown to have abnormal cognitive task-related DLPFC activity (Seminowicz et al., 2011; Mathur et al., 2015). The SPL is a polymodal region thought to exert top-down control on nociceptive reflexes in the brainstem (Liu and Ronthal, 1992; Zambreanu et al., 2005). The STG and MTL regions are also known as the superior temporal polymodal area. This area receives polymodal input from primary and secondary sensory cortices, as well as the insula, and has dense reciprocal projections to the prefrontal cortex and limbic regions, including the anterior and posterior cingulate cortex, the entorhinal cortex and the parahippocampal cortices (Lewis et al., 2000). Polymodal association cortices are generally thought to have a top-down modulatory effect on sensory input.

Therefore, the observed group by stimulus intensity interactions in pain-related activity suggests modulatory networks may be dysfunctional, but these only emerge at higher stimulus intensities. Importantly, patients' pain ratings and stimulation temperature were slightly higher than those of controls. However, group-by-pain intensity interactions in these regions remained significant after controlling for pain intensity ratings, indicating that the observed altered processing during moderate pain in patients may be related to disease-driven changes in modulatory networks. This is further corroborated by our finding that patients showed greater contralateral mINS activity than controls in the moderate pain stimulus (Figure 3). Given that this region is commonly associated with nociceptive processing (Duerden and Albanese, 2013), it is plausible that the nociceptive drive at higher temperatures is not effectively modulated in patients. In line with our findings, several other groups have reported migraine patients have increased trigeminal nociceptive processing in the brainstem (Moulton et al., 2008; Stankewitz et al., 2011) and temporal pole (Moulton et al., 2011). However, these studies were limited to investigating the trigeminal system. Our study expands on this by showing that these abnormalities in nociceptive processing are not restricted to the trigeminal system.

The pain experience is subjective, and shaped by individual factors. Subjects' pain cognitions can alter their perceptual experience (Villemure and Bushnell, 2002). Pain catastrophizing is a well-validated measure of maladaptive thinking patterns related to pain (Sullivan et al., 1995). We found that pain catastrophizing was correlated with greater pain-related activity in bilateral aINS. The aINS has many putative functions (Kurth et al., 2010; Yarkoni et al., 2011; Uddin et al., 2014) including modulatory, homeostatic, and integrative roles (Moayedi, 2014). Similar correlations as those reported here have been identified: pain-related aINS activity and pain catastrophizing have been observed in healthy subjects (Seminowicz and Davis, 2006). Therefore, pain cognitions and nociceptive processing overlap in the aINS. Given that patients have higher catastrophizing scores, it is feasible that nociceptive processing is modulated by these pain cognitions in migraine patients.

We also found that activity in key nodes of the default mode network (DMN; Buckner et al., 2008)—the mPFC and PCC—were negatively correlated with pain catastrophizing. Several studies have reported abnormal activity of DMN nodes in chronic pain disorders (Baliki et al., 2008; Napadow et al., 2010; Davis and Moayedi, 2013; Otti et al., 2013; Kucyi et al., 2014; Ceko et al., 2015). Deactivation of the mPFC and PCC during nociceptive stimulation has been associated with the attentional capture of pain (Kucyi et al., 2013), which is enhanced by negative pain cognitions, such as catastrophizing (Sullivan et al., 1995). This suggests that in the current investigation, patients with high catastrophizing scores may have increased attentional capture by pain and thus an inability to divert their attention away. This may result in a decreased ability to modulate their pain. Taken together, pain-related brain activity in migraine is related to interindividual differences in pain cognitions.

This is an important finding as it provides a novel and feasible therapeutic target. Pain catastrophizing is a significant predictor of migraine clinical severity including chronicity (Radat et al., 2009), and disability (Holroyd et al., 2007). Additionally, changes in pain catastrophizing preceded changes in clinical pain and experimental pain sensitivity in fibromyalgia (Campbell et al., 2012). Furthermore, a recent study showed that pain catastrophizing can be targeted to modulate nociceptive processing and reduce central sensitization in a healthy population (Salomons et al., 2014). Finally, pain interventions decrease pain catastrophizing and mediate corresponding improvements in clinical outcomes (Smeets et al., 2006). Therefore, pain cognitions can serve as a clinical target, and self-report measures of pain catastrophizing can serve as a useful clinical assessment tool.

Finally, we tested whether indices of migraine severity, including headache pain intensity and frequency and disease duration, were related to pain-related activity and white matter structure. Migraine pain intensity over the past month was positively associated with pain-related activity in right pINS and bilateral aINS, and negatively associated with mPFC and PCC activity. These findings suggest that the abnormalities observed in nociceptive processing may be driven by migraine severity. Longitudinal studies are needed to test this possibility.

Migraine frequency was positively associated with pain-related activity in bilateral pINS and increased white matter FA near the somatosensory and motor cortex, suggesting a link between increased attack frequency and enhanced sensory processing of acute noxious stimuli. The inclusion of patients with high frequency migraine in this sample may have increased sensitivity to detect this relationship that has not been previously reported in studies that examined the relationship between frequency and pain-related activity in migraine. Rather, among patients with migraine, frequency has been associated with increased pain-related activations in regions associated with modulatory brain regions such as the DLPFC (Schwedt et al., 2014). Similarly, another study reported that resting state cortical connectivity to the periaqueductal gray is related to migraine frequency (Mainero et al., 2011). Together, our study shows that increased nociceptive drive in migraine is associated with increased sensory processing, in addition to previous findings of disrupted descending modulatory networks.

There are certain limitations to the current investigation. First, we used of a cross-sectional design. Prospective studies that investigate changes in attack frequency over time could provide further insight on potential sensitizing effects of recurrent intense pain as well as increasing attack frequency over time on the central nervous system. Second, our sample size is relatively small—larger studies are needed to replicate and extend the current findings among high-frequency migraine patients. Positive correlations between regions of the insula and disease severity exceeded our cluster forming threshold, but did not survive correction for multiple correction. Therefore, these correlations should be interpreted cautiously and future studies should include a larger sample size of high-frequency migraine patients to increase the power to detect and replicate these findings. Another potential limitation of this study is that we specifically chose to investigate whether patients with migraine had abnormal nociceptive processing outside of the trigeminal innervation. Several prior investigations have examined trigeminal nociceptive processing abnormalities in migraine, and future work should compare stimulation to the face or head and distant body areas. All patients were on medications to manage their migraines, therefore we cannot rule out the possibility that medication effects could contribute to group differences in pain processing. Medication use and history are important clinical variables that may indeed contribute to pathological alterations seen in high-frequency migraine as well as all chronic pain populations. Finally, all participants were engaged in a simple task during pain and this could have affected results, although we showed that pain ratings were not affected by task performance.

In summary, migraine patients with frequent attacks have abnormal extra-trigeminal pain processing related to pain intensity and disease severity. Indices of disease severity were also related to white matter structure. Together, these findings suggest migraine patients have enhanced extra-trigeminal nociceptive processing and disrupted modulatory networks in response to pain. Many of these functional and structural abnormalities occur in the insula, which may be an important therapeutic target for future research.

## Author contributions

DS and MG designed the study. SK and MK collected the data. VM, MM, and CH analyzed the data. DS, VM, and MM wrote the manuscript.

### Conflict of interest statement

The authors declare that the research was conducted in the absence of any commercial or financial relationships that could be construed as a potential conflict of interest.
